# Functional Roles of *O*-Glycosylation

**DOI:** 10.3390/molecules23123063

**Published:** 2018-11-23

**Authors:** Isabelle Breloy, Franz-Georg Hanisch

**Affiliations:** 1 Department Natural Sciences, University of Applied Sciences Bonn-Rhein-Sieg, D-53359 Rheinbach, Germany; isabelle.breloy@h-brs.de; 2 Institute of Biochemistry II, Medical Faculty, University of Cologne, Joseph-Stelzmann-Str. 52, 50931 Köln, Germany

*O*-Glycosylation in general has impact on a diversity of biological processes covering cellular aspects (targeted transport of glycoproteins), molecular aspects (protein conformation, resistance to proteolysis), and aspects involved in cellular communication (cell-cell and cell-matrix interaction). The latter play crucial roles in human infection by viral or bacterial pathogens (innate immunity) and in the progression of cancer.

Mucin-type *O*-glycosylation*—*This more abundant type of *O*-glycosylation is of striking importance in anti-cancer strategies of epithelial tumors involving designed mucin glycopeptides for active specific immunization. In particular, aberrant glycosylation of oncogenic MUC1 has been shown to enhance antigen binding affinity and to reverse tolerance to cytotoxic lymphocytes [1]. Moreover, not only the structures of *O*-linked chains were revealed as critical, but also the sites and densities of peptide modification. This latter aspect became into the focus, when studying the fate of *O*-linked glycans in immunocompetent cells, like dendritic cells. It was found that these modifications were not removed during immunogenic processing and controlled efficiency and specificity of proteasomal and endosomal processing in the MHC class I and II pathways [2,3,4].

Altered *O*-glycosylation of MUC1 is one of the striking features of the oncogenic mucin and functionally involved in its tumor promoting effects. Evidence was revealed in a number of previous studies that cancer-associated glycoforms of MUC1 contribute to progression, invasion and metastasis of epithelial tumors [5]. Besides MUC1 also other mucins, like MUC4 and MUC16 exert pro-tumorigenic effects, partly mediated via their altered *O*-glycosylation and down-stream effects on cell signaling [6]. Communication of cancer cells and lymphocytes in their micro-environment involves the interaction of cancer-associated glycans with lymphocytic lectins [7]. 

Previous work of Reis and coworkers had revealed direct insight into the complex interplay between the human host and *Helicobacter pylori*, as one of the driving forces in the development of gastric cancer [8]. The antagonistic effects of mucin-type *O*-glycans in the control of *Helicobacter* growth were recently reviewed [9]. In this special issue Reis and coworkers provide information on a new gastric cancer model based on a high-throughput 3D spheroid culture methodology that will certainly promote studies of cancer-associated alterations on the proteomic and glycomic levels [10]. The authors demonstrate that their model is superior to conventionally grown cancer cells, as profiles of *O*-glycosylation show culture-dependent differences when comparing 3D with 2D techniques. Cancer cells grown as spheroids developed physiological features that were more authentically reflecting those of the original tumors. Analyzing the glycocalix of four different gastric tumor cell lines under these conditions an increased expression of cancer-associated glycan antigens, like sialyl-Lewis-a and -x and an overall increase in branched glycan structures became obvious.

Other types of *O*-glycosylation—In this special issue Sabine Strahl and coworkers are focusing on the more rare *O*-mannosyl-glycans [11], which had recently been shown to play roles in cadherin-mediated cell-cell contacts [12]. They applied tandem fluorescent protein timer reporter to determine the impact of *O*-mannosylation on protein dynamics. Changes in protein abundance were monitored in parallel to the steady-state protein stability of 137 *O*-mannosylated proteins in *Saccharomyces cerevisiae* [11]. Analysis of these tFT-fusion proteins in three *O*-mannosyltransferase-mutant cell lines revealed that *O*-mannosylation might increase as well as decrease protein stability and abundance. This confirms earlier reported diverse effects of *O*-mannosyl-glycans.

The substoichiometric modification of proteins with *O*-GlcNAc is a highly dynamic *O*-glycosylation of nucleo-cytosolic proteins that plays a crucial role in the regulation of many biological processes similar to protein phosphorylation and is therefore an important aspect in this issue. The group of Tetsuya Okajima analyzed these glycans by mass spectrometry on mouse and *Drosophila* EGF-like repeats [13]. For the first time they could show that *O*-GlcNAc on *Drosophila* Notch1 can be elongated like the mammalian protein when expressed in HEK293-cells, a fact that reveals a structural conservation of this protein during evolution. Showing that only some of the domains on mouse Notch1 are modified exclusively with a single *O*-GlcNAc residue, the authors also concluded that *O*-GlcNAc might impact structure and function of the Notch1-protein.

The role of intracellular protein *O*-GlcNAcylation during the cell cycle was analyzed by Tamas Nagy and his coworkers using double thymidine block [14]. They could show that *O*-GlcNAc is elevated within the short time-frame of mitotic telophase which indicates a potential role of *O*-GlcNAc in the coordination of the cell cycle.

Besides original research papers on this high-impact topic, the current special issue refers in two reviews to functional aspects of protein *O*-GlcNAcylation. Jin and coworkers provide a more comprehensive and broad review of intracellular biological functions of this modification [15], whereas Lefebvre and coauthors refer specifically to the influence of *O*-GlcNAcylation on the biosynthesis of complex glycans and provide a summary of evidences that support a connection between these glycosylation forms [16].
